# Taking Others as a Mirror: Contingent Social Comparison Promotes Task Engagement

**DOI:** 10.3389/fnhum.2018.00476

**Published:** 2018-11-28

**Authors:** Lei Wang, Xiaoshuang Zhang, Lu Li, Liang Meng

**Affiliations:** ^1^School of Management, Zhejiang University, Hangzhou, China; ^2^Neuromanagement Lab, Zhejiang University, Hangzhou, China; ^3^School of Business and Management, Shanghai International Studies University, Shanghai, China; ^4^Laboratory of Applied Brain and Cognitive Sciences, Shanghai International Studies University, Shanghai, China

**Keywords:** social comparison, task engagement, stimulus-preceding negativity, error-related negativity, event-related potentials

## Abstract

Social comparison implemented in an informational while not controlling manner can be motivating. In order to directly examine the effect of contingent social comparison on one’s task engagement, we manipulated social comparison in an experimental study and adopted an electrophysiological approach to measure one’ task engagement. In this experiment, we engaged the participants in a modified stop-watch (SW) task which requires a button press to stop the watch within a given time interval and instructed the participants to either play alone or simultaneously play with a same-sex counterpart. In the latter case, they could freely solicit feedback on their counterparts’ performance besides their own. Enlarged stimulus-preceding negativity (SPN) and error-related negativity (ERN) were observed in the two-player condition, indicating strengthened anticipatory attention toward the task-onset stimulus at the pre-task stage and enhanced performance surveillance during task execution. As a complement, self-report data suggested that the participants were more intrinsically motivated to engage in the SW task when contingent social comparison was present. Thus, converging electrophysiological and behavioral evidences suggested the pivotal role of contingent social comparison in promoting self-directed task engagement.

## Introduction

In our daily life, individuals frequently encounter social comparison, a core feature and shared characteristic of social groups. Social comparison refers to a central mental process, through which people get to compare their own abilities and opinions with those of others for self-improvement and/or subjective well-being (Festinger, 1954; Wills, 1981). A series of studies suggested that self-improvement can be prompted by social comparison, which would subsequently increase the intrinsic reward when performing the original task (Wayment and Taylor, 1995; Suls and Wheeler, 2000). In support of this argument, several functional Magnetic Resonance Imaging (fMRI) studies reported that ventral striatum, which is responsible for reward processing, would show enhanced activation when social comparison information was provided (Fliessbach et al., 2007; Dvash et al., 2010; Bault et al., 2011; Lindner et al., 2014; Simon et al., 2014). While these studies suggested social comparison to be beneficial, some classical behavioral experiments consistently reported the withering effect of social comparison on one’s motivation to perform subsequent tasks (Deci et al., 1981; Vallerand et al., 1986; Jagacinski and Nicholls, 1987; Clinkenbeard, 1989). Based on these conflicting results, we can see that although information provided by social comparison can be beneficial, it is not always conductive to one’s (autonomous) motivation. Thus, a sound theoretical framework is needed to integrate these seemingly contradictory findings.

According to self-determination theory (SDT), one of the most predominant theories on human motivation, there exists three basic psychological needs, which are autonomy, competence and relatedness, respectively (Deci and Ryan, 1985). Once these basic needs are satisfied, people would be more autonomously motivated and then proactively engage themselves in tasks (Deci et al., 2001; Gagné and Deci, 2005; Stone et al., 2008). Compared with intrinsic motivation which serves as a psychological factor in regulating human beings’ behaviors, task engagement is more externally visible and can be measured in a more objective manner (Ainley, 2012; Reeve, 2012). Much previous research has found that intrinsically motivated employees inclined to exhibit higher degrees of work engagement (Gagné and Deci, 2005; Rich, 2006; Thomas, 2009; Haivas et al., 2013; Stoeber et al., 2013). If the fundamental psychological needs mentioned in SDT were satisfied, employees would be more autonomously motivated and then proactively engage themselves in their work (Stone et al., 2008). When it comes to social comparison, information provided by social comparison may serve to facilitate one’s perceived competence and can be beneficial (Ryan and Deci, 2017), but the way that social comparison is implemented may undermine one’s perceived autonomy and counteract its own positive effect. For instance, when interpersonal context is pressured, autonomy would be threatened, which is detrimental to one’s intrinsic motivation (Reeve and Deci, 1996). Thus, social comparison that is implemented in an informational while not controlling manner would be motivating. In this study, we explore the positive effect of contingent social comparison on one’s task engagement, wherein social comparison is encouraged rather than enforced. With contingent social comparison, the information about how well one has performed and the opportunity to compare oneself with others can be provided upon request, which means that the social comparison information is provided only in a voluntary manner. Thus, the participants are not forced to compared with others if they are not willing to. In this study, we pay special attention to the extent to which the participants would proactively engage in the stop-watch (SW) task and the degree of cognitive effort they would voluntarily expend during task execution, that is, one’s self-directed task engagement under contingent social comparison.

In order to measure one’s task engagement, we modified a classical SW task widely adopted by previous studies (Murayama et al., 2010; Ma et al., 2014; Jin et al., 2015; Fang et al., 2018). In a pioneering study, Murayama and co-authors found the game-like SW task, a both challenging and attractive task, was applicable to the measurement of one’s intrinsic motivation (Murayama et al., 2010). Since intrinsic motivation has been suggested to be a driving force of task engagement (Thomas, 2009), we deemed that the SW task would also be appropriate for the purpose of this study. In order to engage participants in social interactions, we employed a two-player online version of the SW task, which was developed in one of our recent studies (Meng et al., 2016). To make sure that the participants were autonomously engaged in the tasks rather than externally driven, they received fixed payments irrelevant to their task performances. Two experimental conditions were set up for each participant, wherein one either completed the SW task alone and only got his/her own task performance (single-player SW task) or played along with a same-sex participant (two-player SW task). In the latter case, one could freely choose whether to solicit feedback on his/her counterpart’s task performance or not after completing the task. In order to objectively measure one’s task engagement during the SW task, we adopted the electroencephalogram (EEG) with high temporal precision. Electrophysiological responses of the paired participants were recorded throughout the experiment.

With the development of cognitive neuroscience, researchers embarked on exploring the neural correlates of intrinsic motivation (Jin et al., 2015). Pioneering electrophysiological studies adopted magnitudes of feedback-related negativity (FRN) loss-win difference wave (d-FRN) upon feedback (Ma et al., 2014; Meng and Ma, 2015) and stimulus-preceding negativity (SPN) toward feedback (Meng and Ma, 2015; Meng et al., 2016; Ma et al., 2017) to measure one’s intrinsic motivation, both of which were agreed on to be sensitive to one’s motivation level (San Martín, 2012; Wang et al., 2017, 2018). Although these pioneering findings are illuminating, engagement during the task was not measured. One motivational stage that interested us was task preparation. Since completion of the SW task naturally requires concentration, the participants have to be well prepared during the pre-task stage and stay focused in order to win. In addition, as our previous studies showed (Ma et al., 2014, 2017; Meng et al., 2016), the participants generally learnt about their task performances immediately upon button press in the SW task, which made it possible for us to measure one’s performance monitoring during the task. Thus, in this study, we focused on cognitive preparation and performance monitoring of the SW task and examined the SPN elicited by the anticipation of task onset stimuli and error-related negativity (ERN) observed around behavioral responses during task execution.

SPN is an event-related potential (ERP) component that reflects processes related to anticipatory attention (Böcker et al., 2001; van Boxtel and Böcker, 2004; Brunia et al., 2012; Meng and Ma, 2015; Meng et al., 2016; Ma et al., 2017; Wang et al., 2017, 2018), which is generally a sustained, negative shift that occurs when a person actively anticipates the onset of certain task-relevant stimuli (van Boxtel and Böcker, 2004). Previous studies have found that task engagement was associated with attention resource availability and that enhanced task engagement could increase participants’ attention level (Matthews et al., 2010a,b). Thus, we adopted the magnitude of SPN to measure one’s task engagement. While most studies focused on the SPN toward feedback stimuli (Meng and Ma, 2015; Meng et al., 2016; Ma et al., 2017), few studies paid attention to the anticipation of task-onset stimuli. According to recent literatures, the SPN can also be observed prior to stimuli that convey instructions for the impending task, whose amplitude would be relatively small (around 1 μV). During this period, the participants should be cognitively preparing for the upcoming task, and magnitude of the SPN can reflect their concentration level (Böcker et al., 2001; van Boxtel and Böcker, 2004; Brunia et al., 2012). In this study, we focused on the SPN elicited during the task preparation stage, a stage before the display of the stopwatch icon. As an effort-requiring task, SW requires millisecond-level precision. Thus, the participants have to be mentally prepared for the task-onset cue in order to better complete it. If contingent social comparison (the opportunity to check one’s counterpart’s task performance in a voluntary manner) is indeed beneficial, we predicted the participants to be more focused when preparing for the upcoming SW task and pay more sustained anticipatory attention toward onset of the task, eliciting a more pronounced SPN at the pre-task stage of the two-player SW task condition (Böcker et al., 2001; Kotani et al., 2015; Meng and Yang, 2018).

ERN is generally elicited within 100 ms of one’s incorrect responses, which directly reflects the level of performance surveillance during the task and helps individuals to improve subsequent behaviors and get better outcomes (Ullsperger et al., 2014). In addition to SPN, previous studies also suggested ERN to reflect the level of task engagement and/or concern about the outcome of a certain task (Tops et al., 2006; Meng and Yang, 2018). It was found that, when people were engaged in certain tasks to a greater extent, they would care more about the commission of errors and react more intensely once they missed a certain goal (Santesso et al., 2005; Tops et al., 2006; Meng and Yang, 2018). In this study, the participants could learn about their task performances when working on the SW task, as they could observe the time point they responded and compare it with the target. It is worth noting that, although we had predefined a success time interval (2.95 s–3.05 s), the participants would naturally compare their performances with the target time point (3.00 s). As most responses would deviate from the target time point to a certain extent, we predicted to observe the ERN despite the objective correctness of the response. According to our hypothesis, if contingent social comparison indeed has positive effects on task engagement, the participants should care more about committing errors or underperforming in the two-player condition, which leads to a more negative ERN during task execution.

## Materials and Methods

### Participants

This study was approved by the internal review board of Zhejiang University Neuromanagement Lab. In order to obtain a representative sample, the participants were randomly selected from students who voluntarily registered for this experiment in response to our message posted on the internal Bulletin Board System (BBS) of Zhejiang University. For each experimental session, two same-sex participants, who were unknown to each other, were recruited and paired. In total, 24 healthy registered graduate and undergraduate students (14 males) in varied majors were enrolled. Data from three participants were excluded due to insufficient valid trials, and ages of the remaining subjects were between 19 and 25 (21.62 years ± 1.50 SD). According to self-reports acquired before the experiment, all of them had either normal or corrected-to-normal vision and no history of neurological disorder or mental diseases. A written informed consent statement was acquired from each participant prior to the experiment.

### Experiment Stimuli and Procedure

Before the experiment, the paired participants met and were briefly introduced to each other at the laboratory. They were then led to take seats in separate rooms and read the instruction printed on paper handouts. The room was dimly lit, sound-attenuated and electrically shielded. Stimuli were presented at the center of a computer monitor with 1-m distance away from the participant and with a visual angle of 2.89° × 3.04°. Each participant should accomplish SW tasks of two different versions, namely, a single-player task and a two-player online task. The former task was a modification of Murayama et al.’s (2010) paradigm, while the latter task was originally developed by Meng et al. (2016). There were two blocks for each version of the SW task and each block contained 40 trials. In order to eliminate the sequence effect, experimental conditions were counter-balanced across the participants. Half of the participants completed the single-player SW task at first and then the two-player version, while the rest participants completed them in a reversed sequence. All the participants were instructed to use a keypad to respond throughout the experiment.

At the beginning of each trial, a fixation cross was displayed for 500 ms at the center of the screen, followed by a 1,000 ms blank screen. Afterwards, the stopwatch icon would appear and automatically start running from 0.00 s. The participants were informed to respond with their dominant hand to stop the watch around 3.00 s by pressing any button on the keypad. They were encouraged to respond as accurate as possible, and the success interval was predefined as 2.95 s to 3.05 s. During feedback, if the participant succeeded, his/her performance would appear in green. If not, in red. The target stopwatch stimulus was displayed for a maximum of 5,000 ms. If the participant did not respond within the given interval, the watch would automatically stop at 5.00 s.

**Figure 1:**
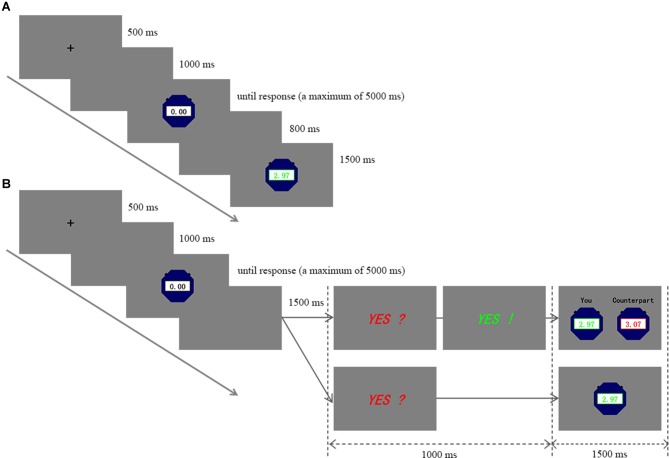
Experimental procedure. The participants were instructed to accomplish the single-player stop-watch (SW) task and the two-player online SW task respectively in two different experimental sessions. For each trial, “+” cue was followed by a 1,000 blank screen. Afterwards, the task-onset cue, namely, the SW icon would appear. Regardless of the experimental condition, the participants were instructed to press any single button on the keypad with their dominant hand to stop the watch around 3.00 s as accurate as possible. **(A)** During the single-player SW task, only the participant’s own performance was displayed after a short delay. **(B)** There was an additional choosing phase following the button response during the two-player online SW task. After both participants responded or the watch stopped automatically, the probe “*YES?*” colored in red appeared. If the participant solicited to view his/her counterpart’s outcome, he/she should respond with button “1” within 1,000 ms, and then the probe would turn into a green “*YES!*” and performance of both players would be displayed. The default option was not to view the counterpart’s performance. If the participant did not respond in time, then only his/her own performance would be shown. For both tasks, the feedback stage would last for 1,500 ms and the between-trial interval would last for 800–1,000 ms.

Just as what Figure 1A illustrated, during the single-player SW task, the participant’s response (or the stop of the watch) was followed by the feedback of his/her own task performance. The major difference between the single-player and the two-player SW task lies in the choosing phase (see Figure 1B). As the procedure of the experiment has to be balanced between the two participants during the two-player online SW task, only after both participants responded would they be directed to the choosing phase of the task. Otherwise, the one who responded earlier had to wait for the counterpart. After both participants responded (or the watch stopped automatically), a probe stimulus “*YES*?” colored in red would appear on the screen. At this stage, each participant could freely choose whether to view the counterpart’s outcome or not. They were assured that the choice was independently made, and neither of them would know their counterpart’s choice. It is worth noting that the decision of a certain player did not influence the feedback information that the other player would receive. For instance, if a player solicited feedback on his/her counterpart’s performance while the counterpart did not do so, only the former player would receive feedback on the task performances of both players in that trial. Compared with previous studies involving some players actually played by the experimenters (Meng et al., 2016; Ma et al., 2017), this study allowed the participants to complete tasks simultaneously with an actual same-sex counterpart and to interact with each other during the tasks. Besides, we did not manipulate feedback information, and the displayed outcomes reflected actual task performances.

In order to match the single-player SW condition, the default option of the two-player SW condition was not to check the counterpart’s task performance. If a participant decided to solicit the counterpart’s outcome, he/she should press button “1” on the keypad within 1,000 ms after onset of “*YES?*.” Upon button press, the stimulus would turn to a green “*YES!*,” and then task performances of both players would be provided for this player. If a participant did not take an active action within 1,000 ms, only his/her own outcome would be presented, as was the case in the single-player SW condition. For both single-player and two-player SW tasks, the feedback stage would last for 1,500 ms. Besides, there was a between-trial interval that lasted for 800–1,000 ms before the next trial started. During the whole experiment, stimuli, recording triggers and behavioral responses were presented and recorded by E-Prime 2.0 (Psychology Software Tools, Pittsburgh, PA, USA).

Before the formal experiment started, each participant was required to practice the single-player SW task for at least 10 trials until he/she thought that it was ready for him/her to start. Also, all the participants were confirmed that they would receive a 40 RMB reimbursement for their attendance, and that their task performances had nothing to do with their payoffs. They were encouraged to stop the watch at 3.00 s as accurate as possible and to enjoy the game. After the experiment, they were debriefed and paid accordingly. Besides, they were instructed to rate their interests in both the single-player and the two-player SW task using a six-point, semantic differential scale (0 = the least interesting, and 5 = the most interesting). Their motivation to win (0 = the weakest motivation, and 5 = the strongest motivation) and the effort they had made (0 = the least effort having made, and 5 = the greatest effort having made) were also measured.

### EEG Recording

EEGs were recorded (band-pass 0.05 Hz to 70 Hz, sampling rate 500 Hz) from 64 scalp sites with the Neuroscan Synamp2 Amplifier (Scan 4.5, Neurosoft Labs, Inc., Sterling, VA, USA). An electrode located between FPz and Fz on the forehead was used as a ground electrode. The left mastoid was selected for the online reference and data of the average of left and right mastoids served as the offline re-reference. Vertical electrooculogram (EOG) was recorded from the electrodes above and below the left eye, and the horizontal EOG was recorded at the left and right orbital rim. The experimenters made sure that electrode impedance was reduced to less than 5 kΩ before the experiment formally started, which was maintained during the whole experiment.

During the offline EEG analysis, the re-reference was conducted by Neuroscan 4.5 while the rest analyses were conducted by Letswave toolbox (Mouraux, Brussels, Belgium^1^) embedded in Matlab (MathWorks, Natick, MA, USA). The vertical EOG artifacts were removed, which was followed by band-pass filtering (0.1–30 Hz for the SPN, and 0.5–30 Hz for the ERN; 24 dB/octave). In terms of the SPN, we segmented the time window of 800 ms prior to stopwatch stimulus onset, with the activity from −800 ms to −600 ms serving as the baseline. For the ERN, the time window of 400 ms before and 400 ms after button press (which would stop the watch) of the participants was segmented and the whole epoch was corrected relative to the baseline, that is, 400–200 ms before button press. Trials containing amplifier clipping or bursts of electromyography activity, as well as whose peak-to-peak deflection exceeded ±100 μV were all excluded. For each participant, the recorded EEGs were separately averaged over each recording site under each condition. For the SPN, the EEG epochs were averaged for single-player (no social comparison) and two-player (contingent social comparison) conditions. For the ERN, there was another within-subject factor, and the epochs were averaged for outcome (success vs. failure) in addition to social comparison (single-player vs. two-player) conditions.

### Data Analysis

Most studies on the SPN reported a right hemisphere dominance (Brunia et al., 2000, 2011; van Boxtel and Böcker, 2004; Kotani et al., 2015; Meng et al., 2016; Ma et al., 2017), which means that the most pronounced SPNs were typically observed from the anterior electrodes on the right side. According to these literatures as well as the topographic map of this study, we analyzed the SPN amplitudes from the electrodes F4, F6, F8, FC4, FC6 and FT8 and then used the mean amplitudes within the time window of 200 ms to 0 ms before onset of the stopwatch stimulus to conduct an ANOVA with within-subject factors of social comparison and electrode. In terms of the ERN, in accordance with previous literatures (Gehring et al., 1993; Riesel et al., 2013) and the topographic map of this study, data from six frontocentral electrodes (F1, Fz, F2, FC1, FCz and FC2) went into the statistical analysis. A 2 (social comparison) × 2 (outcome) × 6 (electrode) repeated measures ANOVA was performed on the ERN within the time window of −50 ms to 50 ms around button press. For both the SPN and the ERN, simple effect analyses were conducted if the interaction effect was significant and the Greenhouse-Geisser correction was applied in all statistical analyses when necessary. For behavioral data, average absolute deviations around the target (the absolute value of the difference between the stopping time and the target time point, that is, 3.00 s) were calculated, and paired *t*-test was adopted for statistical within-subject comparisons.

### Results

#### Behavioral Results

Results of paired *t*-tests showed that success rates of the two conditions were not significantly different from each other (*M*_single-player_ = 0.3679, SD = 0.1231; *M*_two-player_ = 0.3976, SD = 0.1183; *t*_(20)_ = −1.618, *p* = 0.121). When feedback on the counterpart’s task performance was available, the participants checked their counterpart’s task performance in 56.13 ± 29.07% trials. However, the percentage of feedback solicitation was not significantly different between success and failure conditions (*M*_success_ = 0.5985, SD = 0.3381; *M*_failure_ = 0.5218, SD = 0.2946; *t*_(20)_ = 1.527, *p* = 0.143).

Results from subjective ratings indicated that the participants deemed the two-player SW task as more interesting than the single-player version, and that they enjoyed the former task to a greater extent (*M*_single-player_ = 2.76, SD = 1.136; *M*_two-player_ = 3.71, SD = 1.007; *t*_(20)_ = −8.771, *p* < 0.001). Moreover, they held a stronger motivation to win during the two-player game (*M*_single-player_ = 3.00, SD = 0.837; *M*_two-player_ = 3.86, SD = 0.964; *t*_(20)_ = −4.954, *p* < 0.001) and thus paid more effort to complete it (*M*_single-player_ = 3.71, SD = 0.902; *M*_two-player_ = 4.05, SD = 0.805; *t*_(20)_ = −2.646, *p* = 0.016).

#### ERPs

**Figure 2:**
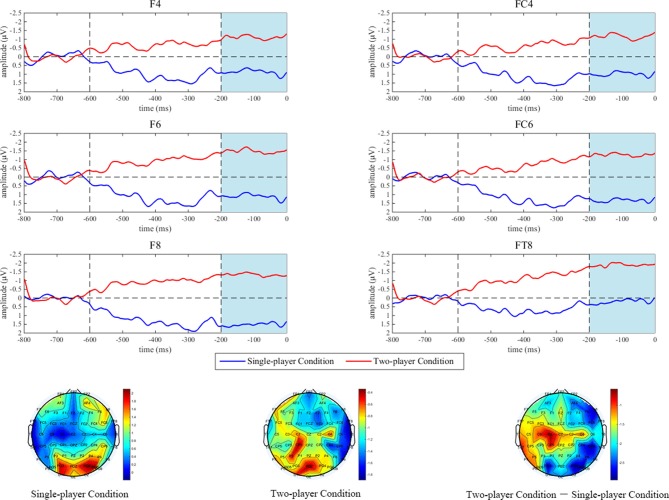
The stimulus-proceeding negativity (SPN) results prior to task onset. Grand-averaged event-related potential (ERP) waveforms of SPN from six anterior electrodes on the right side (F4, F6, F8, FC4, FC6 and FT8) are shown for single-player (marked in the blue line) and two-player conditions (marked in the red line) respectively. The time window of interest is marked in shades of light blue. The scalp topographic distributions of the SPN are provided for the single-player condition (the bar ranges from (−0.20 μV to 2.12 μV), the two-player condition (−2.00 μV to −0.30 μV), and the two-player condition minus the single-player condition (−3.00 μV to −0.55 μV), respectively.

**Figure 3:**
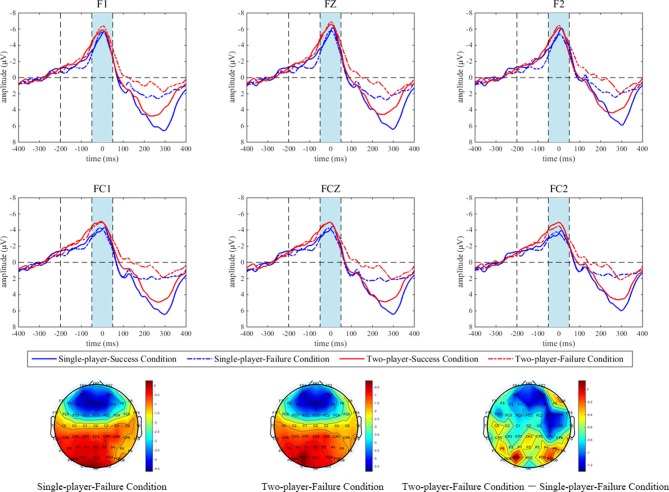
The results of error-related negativity (ERN) around behavioral responses. Grand-averaged ERP waveforms of ERN from six frontocentral electrodes (F1, FZ, F2, FC1, FCZ and FC2) are shown in relation to social comparison (single-player condition vs. two-player condition) and outcome (success vs. failure). Results in the single-player condition and the two-player condition are shown in blue and red lines respectively. In addition, solid vs. dotted lines display outcomes of successes vs. failures. The time window of interest is marked in shades of light blue. The scalp topographic distributions of the ERN are provided for the single-player-failure condition (the bar ranges from −4.65 μV to 0.30 μV), the two-player-failure condition (−5.85 μV to 0 μV), and the two-player-failure condition minus the single-player-failure condition (−1.30 μV to 0.15 μV), respectively.

As shown in Figure 2, the mean SPN amplitude in the single-player condition was 1.0053 μV, while it was −1.3742 μV (negative polarity: smaller voltage value means larger amplitude) under the two-player condition. ANOVA results illustrated a significant main effect of social comparison (*F*_(1,20)_ = 4.570; *p* = 0.045). In spite of this, neither the main effect of electrode (*F*_(2.13,42.55)_ = 1.137; *p* = 0.333), nor the interaction between social comparison and electrode (*F*_(2.88,57.68)_ = 0.620; *p* = 0.599) were significant. For the ERN (see Figure 3), the main effect of social comparison (*F*_(1,20)_ = 7.597; *p* = 0.012) and electrode (*F*_(2.31,46.10)_ = 17.090; *p* < 0.001) were both significant, and the mean amplitudes were greater in the two-player condition (−4.8228 μV) compared with the single-player condition (−3.9734 μV). However, the main effect of outcome was not significant (*F*_(1,20)_ = 0.007; *p* = 0.933). Interaction effects between social comparison and outcome (*F*_(1,20)_ = 0.218; *p* = 0.646), social comparison and electrode (*F*_(3.28,65.57)_ = 0.491; *p* = 0.706), as well as outcome and electrode (*F*_(2.55,51.08)_ = 1.554; *p* = 0.216) were all non-significant. The interaction effect between social comparison, outcome and electrode reached marginal significance (*F*_(3.43,68.53)_ = 2.325; *p* = 0.074).

### Discussion

In our daily life, people are frequently engaged in social comparisons. Through comparing their own beliefs, attitudes, abilities as well as achievements with those of others, people get to understand and evaluate themselves in a better way (Wood, 1996). While the phenomenon of social comparison is frequently observed, it can take place in different forms. Under certain circumstances, social comparison is explicit and may serve as a formal mechanism. For instance, in the work setting, typically the salary of employees is based not only on their objective work performances, but also their performances compared to others. However, in other situations, social comparison may be implicit and contingent, such as the case in the two-player condition of this study. While we emphasized to the participants that their final payoffs were not related with their task performances, they were still confronted with contingent social comparison since information on the counterpart’s performance was available once requested. On the one hand, one can freely choose to learn about the counterpart’s performance at one’s own discretion. On the other hand, without being told, his/her own performance can be solicited by the counterpart at any time during the experiment as well.

Behavioral results of this study suggested that the participants were indeed quite curious about their counterparts’ task performances when contingent social comparison was present, as feedback was requested on 56.13% of the two-player SW trials. According to self-report, the participants were more (intrinsically) motivated to win the game, enjoyed the two-player online SW task to a greater extent, and made greater efforts to complete the task. Since effort provision is closely related to task engagement (Matthews et al., 2002, 2010a), and the participants were not awarded by performance-based rewards, these results jointly indicated that contingent social comparison is beneficial. In line with the behavioral results, the electrophysiological results exhibited that the SPN upon the task onset stimulus loomed larger when contingent social comparison was present (two-player SW task) than when it was absent (single-player SW task). Meanwhile, a similar effect was observed on the ERN. These findings suggested that the participants made a good cognitive preparation and might implement enhanced performance surveillance, which further supported the pivotal role of contingent social comparison in promoting one’s task engagement.

Several mainstream theories on intrinsic motivation and/or social comparison may help explain the current findings from diverse perspectives. According to SDT, external information that is informational while not controlling could effectively enhance one’s intrinsic motivation on a given task through the fulfillment of basic psychological needs (Pittman et al., 1980; Ryan, 1982; Ryan et al., 1983; Koestner et al., 1984; Deci and Ryan, 1985), which may further enhance one’s task engagement (Deci et al., 2001; Gagné and Deci, 2005; Stone et al., 2008). For instance, in the experiment conducted by Koestner et al. (1984), children were randomly distributed into three groups to paint a picture with informational-limits, controlling-limits, or non-limits instructions. Relative to the controlling-limits group, children in the informational-limits group spent more time painting in their spare time and showed greater intrinsic motivation in painting. In this study, feedback on one’s counterpart’s task performance is informational, as it provided information on how well the counterpart was completing the task. This served as a reference and helped one to understand how well he/she was completing the task, which enhanced one’s perceived competence. Besides, this feedback was not implemented in a controlling manner, which reinforced one’s autonomy. For one thing, the participants received fixed payoffs regardless of their task performances. For another, they could choose to receive this additional feedback at their own discretions. Given the informational and non-controlling nature of additional feedback upon request, one’s intrinsic motivation was effectively facilitated, which led to greater engagement in the task (Deci and Ryan, 1985; Stone et al., 2008). According to another argument on social comparison, one’s inclination to compare with others stems from the pursuit of self-improvement, as one may engage in social comparisons to motivate himself/herself to perform better (Suls and Wheeler, 2000). Through social comparison, people get to learn better about their own strengths and weaknesses and thus can actively improve themselves, which brings greater satisfaction and a sense of achievement. Consequently, they will proactively engage themselves in tasks and sustain an optimal status (Wayment and Taylor, 1995). Taken together, as the two-player condition of this study offered an avenue for contingent social comparison, one’s task engagement got enhanced as a result.

Besides verifying the beneficial effect of contingent social comparison on self-directed task engagement, one of the theoretical contributions of this study is the exploration of the neural correlates underlying one’s task engagement during social interactions. In this study, we adopted EEGs with high temporal precision and manipulated the presence of contingent social comparison (the single-player condition vs. the two-player condition). While task engagement has been closely associated with intrinsic motivation (Rich, 2006; Haivas et al., 2013; Stoeber et al., 2013; Reeve and Lee, 2014), existing electrophysiological studies focused on the latter construct, while the neural correlates underlying task engagement were less examined. For instance, in a series of studies, we have applied EEGs to track one’s intrinsic motivation level during effort-requiring tasks and examined various influencing factors of it (Ma et al., 2014, 2017; Meng and Ma, 2015; Meng et al., 2016; Fang et al., 2018). These studies mainly focused on feedback-related cognitive processing, and we adopted either SPN during feedback anticipation or d-FRN during feedback evaluation to measure one’s intrinsic motivation. While these pioneering investigations are inspiring, none of them directly examined proactive task engagement, which could be evidenced by amounts of attention paid to experimental stimuli and the way a participant completed the experimental task (Reeve, 2012). Since cognitive preparation and performance surveillance levels reflect one’s task engagement, through measuring the SPN toward initiation of SW tasks during the pre-task stage and the ERN observed around behavioral responses during task execution, in this study we get to gauge task engagement in a more direct manner.

While these findings are illuminating, we have to recognize that this study is exploratory in nature. While most studies examined the SPN toward feedback or rewards, we focused on the SPN elicited during the anticipation of task-onset stimuli. Given that a few pioneering studies reported to observe the SPN (although relatively small in magnitude) when the participants are cognitively preparing for the upcoming task (Böcker et al., 2001; van Boxtel and Böcker, 2004; Brunia et al., 2012; Meng and Yang, 2018) as is the case in this study, follow-up studies on the SPN observed on the pre-task stage are needed to provide additional support for our current findings. In addition, although online performance surveillance can be implemented, compared with speeded response tasks such as the Stroop task and the Flanker task, the SW is not an optimal task to elicit the ERN. As reported in previous studies, a correct while relative slow response in speeded response tasks would be accompanied by an ERN pattern, which is similar with that elicited by failures (Vidal et al., 2003; Gehring et al., 2012). As a successful attempt in the SW (such as stopping the watch at 3.04 s) still deviates from the target time point (3.00 s) to a certain extent, this might help explain the null outcome/valence effect on ERN in this study. Another limitation of this study is that, while we measured the intrinsic motivation level through self-report, we neglected to include subjective ratings of task engagement in our questionnaire, which might give further support to the electrophysiological findings of this study.

## Conclusion

In order to directly examine the effect of contingent social comparison on task engagement, we modified the classical SW task and instructed the participants to either play alone or simultaneously play with a same-sex counterpart. In the latter case, they have the discretion in deciding whether to view their counterparts’ outcomes in addition to their own or not. The participants reported to enjoy the two-player SW game more and worked harder on it. In addition, compared with the single-player condition, more pronounced SPNs and ERNs were respectively observed at the pre-task stage and around behavioral responses in the two-player condition. Thus, converging evidences suggested that contingent social comparison would effectively promote one’s autonomous task engagement.

## Ethics Statement

This study was carried out in accordance with the requirements of the Neuromanagement Lab Ethics Committee at Zhejiang University. All the participants gave a written informed consent according to the Declaration of Helsinki. All the participants had normal or corrected-to-normal vision. None of them reported any history of psychiatric or neurological disorders.

## Author Contributions

LM, LW and XZ conceived and designed the study. XZ and LL collected and analyzed the data. XZ and LM interpreted the data and drafted the manuscript. LM, LW, XZ and LL reviewed and edited the manuscript. LM and LW administered the project.

## Conflict of Interest Statement

The authors declare that the research was conducted in the absence of any commercial or financial relationships that could be construed as a potential conflict of interest.

## Footnotes

^1^http://www.letswave.org
